# Research on Multimodal Dance Movement Recognition Based on Artificial Intelligence Image Technology

**DOI:** 10.1155/2022/4785333

**Published:** 2022-07-12

**Authors:** Zhuo Zeng

**Affiliations:** School of Xihua University, Sichuan, 610000, China

## Abstract

At present, most robot dances are precompiled. Changing music requires manual adjustment of relevant parameters and metamovements, which greatly reduces the fun and intelligence. In view of the above problems, this paper designed CNN system, studied the multimodal dance movement recognition algorithm of artificial intelligence image technology, and completed the construction of a multimodal dance movement calculation system example. The results show that the CNN algorithm and the Winograd algorithm-based coprocessor-optimized CNN network in multimodal dance movement recognition with image technology reduce from a maximum of 132s to 26s in the runtime criterion, with a maximum reduction of 80%; from a maximum of 73.5% to 16.2% in the memory access criterion, with a maximum reduction of 57.3%; and from a maximum of 93.6% to 25.2% in the power consumption ratio criterion, with a maximum reduction of 68.4%. In the power consumption ratio criterion, the maximum reduction from 93.6% to 25.2% is 68.4%. The maximum accuracy of the proposed optimization method is 95.1%. The solution is proposed to address the problem of insufficient performance of traditional dance movement recognition, which will contribute to the development of artificial intelligence and dance industry.

## 1. Introduction

Dance is one of the oldest forms of traditional artistic expression, with its unique vitality and creativity. As a special product of human civilization, art has a very important role in promoting the development of the whole society and the prosperity of the country. Among them, dance is a very ancient and unique charming art product, which has its own very important relevance and mechanism as one of the carriers of traditional Chinese culture [1–3]. The revitalization of dance can promote the excellent traditional culture and enhance the soft power of the country culturally. In a diversified world, there are tens of thousands of ways to satisfy spiritual needs, and dance, as an elegant plastic art in motion, is one of the most popular ways and also plays a role in people's socialization. Dance, not only a social and cultural form, is also a dynamic art presented in a rich and varied body language. Dance movements include bending; leaning together, extending; gliding; feet drawing circles on the ground; striking; dissolving; drawing circles in the air; rising; drawing up, pulling up; running of hands; the direction of feet and body. With the rapid development of artificial intelligence, dance also begins to enter the era of information and intelligence. How to identify and optimize multi-modal dance movements through artificial intelligence so as to achieve dance in the intelligence to maintain the reality remains a challenge.

**Figure 1:**
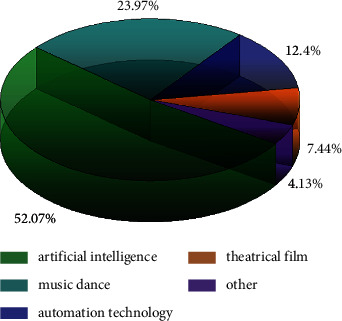
The proportion of dance movement research.

Dance is a special way for people to express their emotions [4, 5], and dance is different from other expressions in that it uses the human body as a tool to express emotions through artistic processing of the human body. Many dances are created in the process of people's work, and they have very strong local characteristics and humanistic feelings. People who express their emotions through dance also carry out the reflection of our socioeconomic conditions, creating many dances rich in local characteristics. Dance relies heavily on the ability of people to synchronize their movements with music in a rhythmic manner. The combination of artificial intelligence and dance has given rise to robotic dance [6–8], which requires the presentation of a continuous, balanced, and aesthetic movement, in addition to a certain level of intelligence required for the dance. Robotic dance has contributed to the development of dance, which is traditionally limited to the human sphere and uses humans as the main dance vehicle. Due to the rapid development of artificial intelligence, robotic dance has subverted people's perceptions to a certain extent. The related research has also drawn wide attention, so we conducted a research related to dance movements in the relevant database, and the search results are shown in Figure 1, which indicates that the main research work on dance movements in computer artificial intelligence accounts for the largest proportion, 52.07%, and the amount of music and dance research, which is ranked second, is 23.97%, followed by automation-related dance research, accounting for of 12.4%, which indicates that research on artificial intelligence for dance movement recognition is extremely important. Bian [2] et al. proposed to validate the generated model by comparing artificial intelligence (AI) output with dance teachers' assessments, where they first elicited from teachers the dance elements they typically focus on for rhythmic assessment (i.e., tempo, pause, step length, and weight transfer). Then, selected features are extracted from raw motion sensor data related to the rhythmic patterns of the learner's dance, their synchronization with the music beat, and specific features of the song being played. Finally, a machine learning (ML) algorithm is used to create a predictive computational model using these features. To validate quantitative comparisons between ML output and dance teachers' assessments of learners' dance performance, and to provide a qualitative analysis of the potential pedagogical uses of ML model output as envisioned by dance teachers. Sun [3] proposed a new approach to the spatiotemporal dynamics of dance using keypoints integrated with GRU networks, creating a video archive of live recordings from different trained dancers and newspaper clippings from the Internet. The Deep Pose Estimator coupled GRU model uses a deep learning pose estimator to process the spatial aspect and a GRU network to process the temporal perspective. The efficiency of the proposed method is compared with benchmark methods such as 3D convolutional neural network-based models, temporally distributed CNN-LSTM [9–12] models, and hybrid migration learning-LSTM models, and the results show that the proposed method outperforms other method resolutions even under different videos. Hui [13] et al. used artificial intelligence to provide a system of virtual dance partners, providing a new direction for research such as artificially intelligent dance movements. Joo-Wha Hong [5] investigated how people perceive AI music generators and evaluate their songs based on the different characteristics they possess, the independent creativity, and humanization characteristics of AI music generators do not affect music evaluation, relying on the theoretical framework of anthropomorphism and creative machine heuristics, and designed a 2  x  2 experiment in which AI's perceptual anthropomorphism (high vs. low anthropomorphism) and its autonomous creativity (independent vs. dependent creativity) were controlled.

The above literature analysis illustrates the versatility and related excellent stability performance of artificial intelligence in the fields of dance recognition, dance education, etc. Robotic dance is an attractive and highly emerging research field. As an elegant and moving plastic art that perfectly combines auditory and visual arts, it breaks through people's understanding of existing traditional entertainment and makes people's life more colorful. However, in the process of transferring the real dance to the commonly used computer network client, due to the variety of dance movements and the inconsistency of the related dance images, there are often problems such as slow image processing speed, high power consumption, and network bandwidth difficulty in the process of extracting information and calculating related data from the dance images; therefore, this paper addresses the problems related to the dance movement image processing. Artificial intelligence method is used to solve and optimize the related problems in dance movement recognition by combining convolutional neural network with Winograd algorithm.

## 2. Concepts Related to Artificial Intelligence Dance Movement Recognition

**Figure 2:**
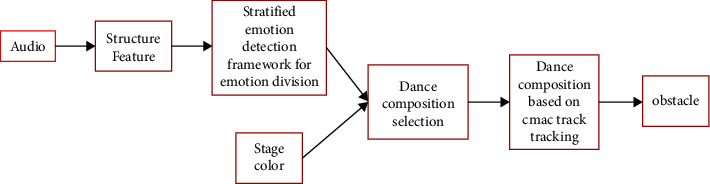
Dance composition flowchart.

Artificial Intelligence (AI), which refers to the intelligence displayed by programs written by humans, etc., is the technology and science of studying and discovering human intelligence [14–16]. Artificial intelligence technologies are now widely used in various production activities, and the most widely used fields mainly include image processing, natural language processing, and human speech and semantic recognition. Image processing can be combined with a variety of application scenarios and has shown excellent performance in several fields. In the process of dance image processing [17–19], the main system flowchart is shown in Figure 2. First, the initial structure of the image such as the audience is divided, and then the emotions expressed by the characters are divided, while at the same time, the color processing contained inside the image is extremely important, and finally in the input in the information processing module [20, 21].

In the process of processing image data by artificial intelligence, there are several main problems as follows:

1. Data transmission is limited by the network. The huge amount of data generated at the edge is uploaded to the cloud for processing, and then the processing results are returned from the cloud, which not only wastes a lot of network resources but also consumes a lot of time and causes a lot of network latency;
2. Data processing is limited by cloud server computing bottlenecks [22]. It is still unable to meet the demand of the exploding data computing and processing. At the same time computing demand in different time periods, there are peaks and valleys, in the peak of demand edge devices to provide data to wait in line for processing, seriously affecting the normal operation of edge devices; in the trough of demand, a large number of computing resources remains idle, resulting in waste
3. There are security and privacy issues in data network transmission [23]. The data generated during image processing involves personal privacy, which is likely to be stolen by hackers through loopholes during network transmission, resulting in personal privacy data leakage.

### 2.1. Feasibility of Artificial Intelligence Technology Applied to Multimodal Dance Movement Recognition

Most of the current robot dances are precompiled, and if you change the music, you need to manually adjust the relevant parameters and metamovements, which is much less interesting and intelligent. If the robot dance is driven not only by the beat of the music but also by the emotion and tension of the music, and it is not just pattern matching, and the interest will be greatly increased. Among them, the demand of dance image processing is one of the main demands of multimodal dance recognition. Image processing usually requires complex operations, and applying artificial intelligence techniques to dance image processing has better results in reducing the amount of operations and improving the performance of image processing. In this paper, an AI-based image recognition multimodal dance movement system is designed to solve the problems of poor real-time, high power consumption, and high network bandwidth requirement of cloud computing system. Convolutional neural network is one of the most widely used and efficient algorithms in the field of artificial intelligence image processing technology. The advantages of convolutional neural networks over other traditional image processing algorithms include the fact that convolutional neural networks can take the original image directly as input without the need for preadjustment of the original image. The CNN training process is mainly divided into forward propagation and backward propagation [24, 25].

**Step 1 .**

**Figure 3:**
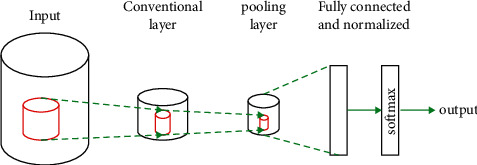
Simplified convolutional neural network structure.

Firstly, the main purpose of the algorithm processing is to extract a certain feature from the image. The schematic diagram of the feature map and convolution kernel involved in the operation is shown in Figure 3.

In the image information processing, first of all, before the convolutional network starts to work, the basic information of the image is input, and then a preliminary information is extracted, including the basic information of the dance movement, the number of dancers and the stage, etc. Through the preliminary, it is simple that processing the information is input into the convolutional calculation.

(1) $$\begin{array}{c} a_{d,i,j}=f\left(\sum_{d=0}^{D-1}\sum_{m=0}^{F-1}\sum_{n=0}^{F-1}w_{d,m,n}x_{d,j+m,j+n}+b\right), \end{array}$$

where *D*, *F* are the number of filters and the size of the convolution kernel, respectively.

**Step 2 .**

In the background conditions of very hot artificial intelligence, the research on hardware acceleration of artificial intelligence algorithms is also getting hotter. Convolutional neural networks are used in many projects as a very effective network structure for image processing. In the convolutional neural network, the convolutional operation occupies more than 80% of the entire convolutional neural network. The data is processed enough to filter out a large number of parameters, improve training accuracy, and reduce errors. Commonly used types of operations are maximum, average, and summation. The basic information of the previous image is weighted, biased, summed, and then outputted by the function, and the basic processing is as follows:

(2) $$\begin{array}{c} y=f\left(w\cdot x\right)+b. \end{array}$$

**Step 3 .**

The information of the image has been extracted and processed basically in step 2, and the next step is to reverse update the information in step 2 to further optimize the results and improve the accuracy and stability of the processing process. The process is divided into the following main processing procedures to achieve high accuracy and stability of image action recognition.

(i) By comparing the feature data of the prediction result in the image recognition process with the feature result data of the actual image, the error exists in it so as to facilitate the next update feedback and further processing:
  (3) $$\begin{array}{c} \delta^{i,l}=-\left(y_{real}\left(i\right)-y_{pre\;di\;ct}\left(i\right)\right)*\sigma \left(a^{i,l}\right), \end{array}$$
(ii) where *y*_*predict*_ is the image prediction data result data, and *y*_*real*_ is the actual feature result data.
(iii) First, the obtained error is initially input:
(iv) By preliminarily transmitting and processing the error calculated in (i), in the process of intelligently processing image action recognition, the formula for feature recognition extraction is as follows:
  (4) $$\begin{array}{c} \delta^{i,l}={\left(w^{l+1}\right)}^{T}\delta^{i,l+1}⊙\delta \left(a^{i,l}\right). \end{array}$$
(v) Then, through the initial input of the error again, it is determined whether the error is too large to meet the requirements of image feature extraction. If it does not meet the set target, it will be eliminated and the information feature extraction will be performed again; if the requirements are met, the next step refined feature extraction processes the results of the first step again:
  (5) $$\begin{array}{c} \delta^{i,l}=\delta^{i,l+1}*rot180\left(w^{l+1}\right)⊙\delta \left(a^{i,l}\right). \end{array}$$
(vi) After the judgment and processing in the second step, the basic information of the image is well guaranteed and basically meets the set requirements.
(vii) Finally, the processing results are processed in more detail.
  (6) $$\begin{array}{c} \delta^{i,l}=\text{upsamples}\left(\delta^{i,l+1}\right)⊙\delta \left(a^{i,l}\right), \end{array}$$
(viii) where *δ*(*a*^*i*,*l*^) module functions that can be handled in more detail. The convolution operation is converted to matrix multiplication, which can rearrange the elements in the feature map, convert the feature matrix into a special matrix, and store it in the memory continuously, which improves the speed of memory reading.
(ix) After the information extraction process described in the previous section, in order to extract and process some more detailed information in the image, we update the weights *w* and bias *b* of the processing process twice, in order to better update and track the relevant image information in real time.

After getting the feedback from the previous images, we update them to get some more detailed body movements, such as hand dancing, foot moving, etc.

(7) $$\begin{array}{c} w^{l}=w^{l}-\alpha \sum_{i=1}^{m}\delta^{i,l}{\left(\alpha^{i,l-1}\right)}^{T}. \end{array}$$

After the feedback, the deviation update for the image is processed using (8) to obtain the subsequent intermediate processing update values.

(8) $$\begin{array}{c} b^{l}=b^{l}-\alpha \sum_{i=1}^{m}\delta^{i,l}. \end{array}$$

Firstly, the neural network algorithm is deployed to the processing module, which collects images or other information in real time through the data acquisition module. At the same time, the collected image or video information is processed and information analyzed using the neural network algorithm. Finally, in the overall processing process, all the information from the previous period is checked and updated again to ensure the accuracy and authenticity of the images as much as possible.

(9) $$\begin{array}{c} w^{l}=w^{l}-\alpha \sum_{i=1}^{m}\alpha^{i,l-1}*\delta^{i,l}. \end{array}$$

After the whole update process to get the relevant update data, the deviation of the image again follows the processing work, through equation (10) reasoning completed in real-time feedback update to the next step.

(10) $$\begin{array}{c} b^{l}=b^{l}-\alpha \sum_{i=1}^{m}\sum_{u,v}{\left(\delta^{i,l}\right)}_{u,v}, \end{array}$$

where *α*^*i*,*l*−1^ is expressed as the output of the *i*-th neuron of the (*l*-1)-th layer.

After an image action has undergone all the processing methods described above, the extraction of relevant feature information has been completed. In order to further optimize the accuracy of the extracted features, we use the image distortion method in the image processing process. The loss function is used as a feedback indicator.

(11) $$\begin{array}{c} J_{c}=-\frac{1}{N}\sum_{1}^{N}\sum_{i=1}^{k}\log\left(y_{c}\left(i\right)\right), \end{array}$$

where *y*_*c*_(*i*) is the predicted value, *y*(*i*) is the actual value, and *N* is the number of samples.

### 2.2. Winograd Algorithm

The Winograd algorithm is a fast convolution algorithm. By finding the common terms in the multiplication of convolution operations and combining some multiplication operations, the multiplication resources required for calculation can be saved. Multipliers consume a lot of resources in digital processors, so reducing multiplication operations can improve certain performance. The specific calculation process is as follows:

(12) $$\begin{array}{c} \left[\begin{array}{cccc} x_{0} & x_{1} & \ldots & x_{n-1}\\ x_{1} & x_{2} & \ldots & x_{n}\\ \vdots & \vdots & \vdots & \vdots \\ \begin{array}{c} x_{m-2}\\ x_{m-1} \end{array} & \begin{array}{c} x_{m-1}\\ x_{m} \end{array} & \begin{array}{c} \ldots \\ \ldots \end{array} & \begin{array}{c} x_{m+r-2}\\ x_{m+r-1} \end{array} \end{array}\right]*\left[\begin{array}{c} w_{0}\\ w_{1}\\ \vdots \\ \begin{array}{c} w_{r-2}\\ w_{r-1} \end{array} \end{array}\right]=\left[\begin{array}{c} y_{0}\\ y_{1}\\ \vdots \\ \begin{array}{c} y_{r-2}\\ y_{r-1} \end{array} \end{array}\right], \end{array}$$

where *m* is the output size and *r* is the convolution kernel size.

In addition, the symbol of *r* input filter is expressed as F, and the minimum number of multiplications required is calculated as follows:

(13) $$\begin{array}{c} \mu \left(F\left(m,r\right)\right)=m+r-1, \end{array}$$

where *μ* (*F*(*m*, *r*)) is the minimum number of multiplications required for calculation.

For the two-dimensional calculation of the subfilter, the formula can be derived from the one-dimensional formula as follows:

(14) $$\begin{array}{c} \mu \left(F\left(m*n,r*s\right)\right)=\left(m+r-1\right)\left(n+s-1\right). \end{array}$$

It can be inferred from the above formula that the minimum number of multiplications is required by CNN calculation after optimization. In addition, in order to further reduce the computational complexity of optimal minimum multiplication, the output length of 2 is selected at this time, and the convolution calculation of the input length of 4 is carried out, and the calculation process is as follows:

(15) $$\begin{array}{c} F\left(2,3\right)=\left[\begin{array}{ccc} \begin{array}{c} x_{0}\\ x_{1} \end{array} & \begin{array}{c} x_{1}\\ x_{2} \end{array} & \begin{array}{c} x_{2}\\ x_{3} \end{array} \end{array}\right]*\left[\begin{array}{c} w_{0}\\ w_{1}\\ w_{2} \end{array}\right]=\left[\begin{array}{c} x_{0}*w_{0}+x_{1}*w_{1}+x_{2}*w_{2}\\ x_{1}*w_{0}+x_{2}*w_{1}+x_{3}*w_{2} \end{array}\right]. \end{array}$$

It can be obtained that 6 multiplications are needed for direct operation, but only 4 multiplications are needed for Winograd. Specific optimization methods are as follows:

(16) $$\begin{array}{c} m_{1}=\left(x_{0}-x_{2}\right)w_{0}, \end{array}$$

(17) $$\begin{array}{c} m_{2}=\left(x_{1}+x_{2}\right)\frac{w_{0}+w_{1}+w_{2}}{2}, \end{array}$$

(18) $$\begin{array}{c} m_{2}=\left(x_{1}-x_{2}\right)\frac{w_{0}+w_{1}+w_{2}}{2}. \end{array}$$

By combining equations (16)–(18), (19) can be obtained as follows:

(19) $$\begin{array}{c} F\left(2,3\right)=\left[\begin{array}{ccc} \begin{array}{c} x_{0}\\ x_{1} \end{array} & \begin{array}{c} x_{1}\\ x_{2} \end{array} & \begin{array}{c} x_{2}\\ x_{3} \end{array} \end{array}\right]*\left[\begin{array}{c} w_{0}\\ w_{1}\\ w_{2} \end{array}\right]=\left[\begin{array}{c} m_{1}+m_{2}+m_{3}\\ m_{2}-m_{3}-m_{4} \end{array}\right]. \end{array}$$

In this way, it takes only four multiplications to complete a calculation that would otherwise require six multiplications, saving two multiplication resources. In fact, it is not difficult to conclude that the larger the base unit used, the more multiplications can be saved. If the dance image you need to identify is large, you can also use a larger base unit. Therefore, customized optimization for different projects can achieve the best optimization effect from the algorithm level.

## 3. Experimental Verification and Comparative Analysis

### 3.1. Comparative Analysis of Accuracy of Multimodal Dance Movement Recognition Results

The aesthetic function of dance, including beautiful musical melody, elegant dance posture, and vivid dance image, can accurately identify dance movements through artificial intelligence image processing technology. Through the gradual artificial intelligence image optimization and feature extraction, it can realize the unification of the beautiful dance posture and beautiful melody in the computer client. Therefore, the multimodal dance movement recognition of artificial intelligence can fully extract the format movements in the dance, integrate them into the dance database, and generate relevant videos, which can also provide learning materials for the cultivation and continuous improvement of the aesthetic awareness and ability of dance learners. As a visual art that conveys emotion, dance is a typical embodiment of human imagination and creativity. It not only has the essence of creation and appreciation but also reflects people's appreciation ability. Therefore, the extraction and present of dance features play an important role in activating people's thoughts, cultivating and developing intelligence, and adjusting people's thinking.

**Figure 4:**
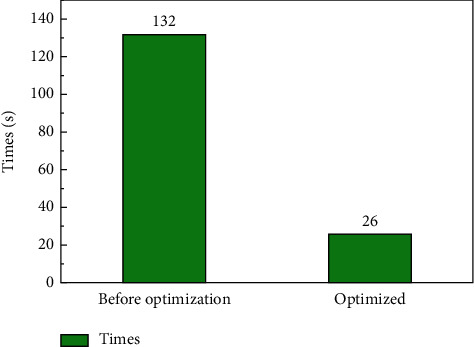
Running time comparison.

In the process of artificial intelligence dance image movement recognition, when the same convolution operation is processed by the CNN network CPU without optimization in the data processing module, a total of 36 times of memory need to be accessed, and 18 times of minimum multiplication and division mathematical operation and 18 times of addition and subtraction mathematical operation need to be carried out. Considering that different compilation options have different optimization effects on the program, the running status of CNN before optimization is analyzed here. Winograd implements the 32 bit single-period multiplier in the execution stage. Considering the zero-cost hardware cycle optimization and ignoring other control signals, it takes at least 4 minutes to complete the convolution operation. As a result, the accelerated CPU achieved more than an eight-fold improvement in performance. The program runtime comparison is shown in Figure 4. The maximum running time is reduced from 132s to 26s, with a maximum reduction of 80%. This shows that the data processing module after optimization is faster for data processing. In the same time, the processing speed after optimization is about 5 times that of the previous, and the processing capacity of the optimized artificial intelligence image processing module has been greatly improved.

**Figure 5:**
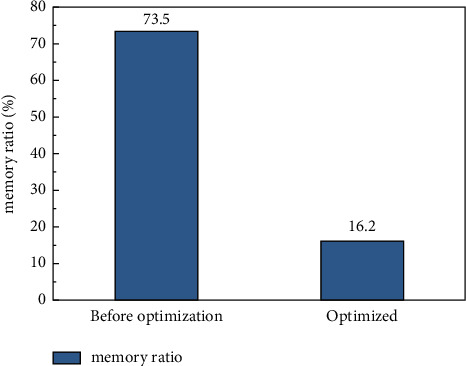
Memory access ratio diagram.

Similarly, in the process of artificial intelligence image processing, the memory ratio of the computer is also a difficulty. On the premise of consistent feature extraction effects for the same image with the processed information extracted, the smaller the memory ratio, the more accurate the feature extraction will be and the more accurate the control of multimode dance movements will be. In our study, the memory access results in the recognition of 105 image actions processed simultaneously are shown in Figure 5. From Figure 5, it can be clearly seen that the ratio of memory in the optimized extraction module decreases from 73.5% to 16.2%. Compared with CNN without Winograd optimization, the number of memory access is reduced by about 57.3%. The optimization used in this paper takes only 2 minutes. However, through ordinary operations (CNN), even excluding the influence of control signals, data activation, and pooling layer computing need a lot of command operations. In this case, the network computing personality can be improved even more by coprocessing.

**Figure 6:**
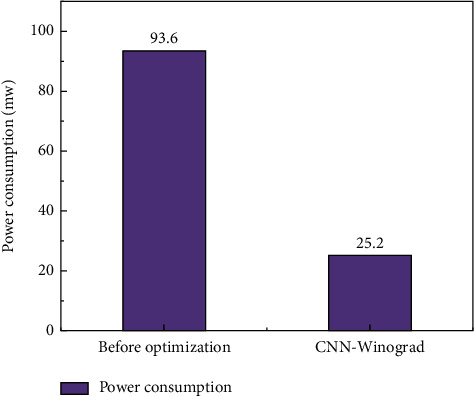
Power consumption performance comparison.

It is worth mentioning that in the intelligent image action recognition, more and more attention is paid to the energy consumption in the processing process. The lower the energy consumption, the lower the processing cost and the higher the economic benefits. Therefore, in this paper, in order to reduce the power consumption during the network operation, we explore how the optimized network can improve the economic efficiency. This paper further optimizes the network operation cost and conducts an accurate statistical analysis of the energy consumption in the image recognition process before and after optimization. The power consumption ratio comparison of network computing is shown in Figure 6. It can be seen from Figure 6 that the power consumption ratio of the CNN network optimized by the Winograd algorithm can be reduced to 1/4 of the original, from the original maximum of 93.6% to 25.2%, with a maximum reduction of 68.4%. The experimental results show that through this special processing, from the algorithm level, the convolution calculation is optimized, hardware resources are saved, and processor performance is improved. In addition, through the optimization of processing time and memory ratio, and then the energy consumption of the intelligent image processing process is directly optimized, the economic performance is improved, and most of the energy consumption is saved.

**Figure 7:**
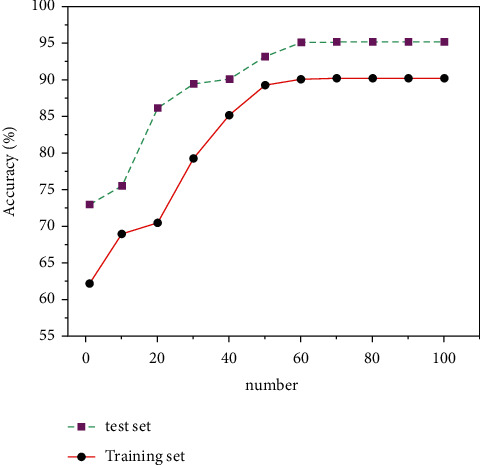
Comparison diagram of algorithm accuracy.

In order to further prove the superior performance of our proposed optimization algorithm in multimodal dance action recognition, we selected 2,000 images to implement our algorithm on MATLAB software, of which 1,600 images were used as training set and 400 images were used as test set. As a test set. The training results are shown in Figure 7. After the optimization of the Winograd algorithm is applied, the training set reaches a state of convergence after 60 iterations, and the accuracy can reach 90.2%. In the test set, the accuracy of about 90% can be achieved after 39 iterations. After 60 iterations, the accuracy can be as high as 95.1%.

## 4. Conclusion

In this paper, we give a basic description of the concept of multimodal dance and neural network algorithm, and introduce the CNN algorithm structure and calculation process for multimodal dance recognition. We study the operation process of convolution operation in CPU. Aiming at the situation that the convolution operation requires frequent memory access and a large number of multiplication operations, a coprocessor hardware acceleration module based on the Winograd algorithm is designed to accelerate the convolution operation. And we compared the pros and cons of the CNN and the CNN optimized based on the Winograd algorithm through three evaluation criteria: running time, memory access times, and performance comparison. The result is shown as follows:

1. The CNN algorithm and the Winograd algorithm-based coprocessor-optimized CNN network in the multimodal dance action recognition of image technology can reduce the running time from a maximum of 132s to 26s, with a maximum decrease of 80%. The judging criteria dropped from a maximum of 73.5% to 16.2%, with a maximum drop of 57.3%. The convolution operation is performed on the feature map by using the operator, and a lot of memory space is saved compared with the operation of converting the convolution operation to matrix multiplication.
2. Power consumption is one of the economic performances that need to be considered in image processing. In the multimodal dance action recognition in image technology CNN algorithm and coprocessor-optimized CNN network based on Winograd algorithm, the power consumption ratio is reduced from a maximum of 93.6% to 25.2% with a maximum reduction of 68.4% in the judging criteria. The same large performance improvement can be obtained by using the operator to perform convolutional operations on the feature map, which in turn produces a direct optimization of the energy consumption of the intelligent image processing process, improving the economic performance and saving most of the energy consumption.
3. The CNN algorithm and the Winograd-based algorithm in multimodal dance movement recognition in image technology can reach 90.2% accuracy in the training set and finally stabilize at 95.1% accuracy in the test set after testing. This shows that our proposed optimization method can maintain good accuracy and stability in the recognition of image movements.

## Data Availability

The dataset can be accessed upon request.
